# Progressive GAA·TTC Repeat Expansion in Human Cell Lines

**DOI:** 10.1371/journal.pgen.1000704

**Published:** 2009-10-30

**Authors:** Scott Ditch, Mimi C. Sammarco, Ayan Banerjee, Ed Grabczyk

**Affiliations:** Department of Genetics, Louisiana State University Health Sciences Center, New Orleans, Louisiana, United States of America; University of Minnesota, United States of America

## Abstract

Trinucleotide repeat expansion is the genetic basis for a sizeable group of inherited neurological and neuromuscular disorders. Friedreich ataxia (FRDA) is a relentlessly progressive neurodegenerative disorder caused by GAA·TTC repeat expansion in the first intron of the *FXN* gene. The expanded repeat reduces *FXN* mRNA expression and the length of the repeat tract is proportional to disease severity. Somatic expansion of the GAA·TTC repeat sequence in disease-relevant tissues is thought to contribute to the progression of disease severity during patient aging. Previous models of GAA·TTC instability have not been able to produce substantial levels of expansion within an experimentally useful time frame, which has limited our understanding of the molecular basis for this expansion. Here, we present a novel model for studying GAA·TTC expansion in human cells. In our model system, uninterrupted GAA·TTC repeat sequences display high levels of genomic instability, with an overall tendency towards progressive expansion. Using this model, we characterize the relationship between repeat length and expansion. We identify the interval between 88 and 176 repeats as being an important length threshold where expansion rates dramatically increase. We show that expansion levels are affected by both the purity and orientation of the repeat tract within the genomic context. We further demonstrate that GAA·TTC expansion in our model is independent of cell division. Using unique reporter constructs, we identify transcription through the repeat tract as a major contributor to GAA·TTC expansion. Our findings provide novel insight into the mechanisms responsible for GAA·TTC expansion in human cells.

## Author Summary

The human genome is comprised of the DNA base sequences used by the cell as a blueprint to direct proper cellular function. Changes in this sequence, known as genomic instability, often interfere with vital cellular functions, resulting in genetic disorders. Repetitive DNA sequences are particularly susceptible to genomic instability. Trinucleotide repeat disorders are caused by three base repeat sequences that increase in size when passed from parent to child and during aging. Trinucleotide repeat expansion results in disease when the size of the repeat sequence increases into the pathogenic size range. Our understanding of the mechanisms responsible for these repeat length changes is incomplete and modeling repeat expansion in human cells has proven difficult. Here, we have developed a unique human cellular model of GAA·TTC trinucleotide repeat expansion, the causative mutation in Friedreich ataxia. Using this model, we characterize GAA·TTC expansion in human cells and identify gene transcription as a key regulator of GAA·TTC repeat expansion. The findings of this study provide novel insight into the mechanisms contributing to trinucleotide repeat expansion in human cells and present new implications for certain therapeutic approaches in Friedreich ataxia.

## Introduction

Trinucleotide repeat disorders are caused by the expansion of unstable tandem repeats to a pathogenic size above disease-specific length thresholds [1]–[5]. Disease-associated trinucleotide repeat arrays include CAG·CTG, CGG·CCG, and GAA·TTC sequences. Disease pathology in these disorders is often progressive and usually involves a neurodegenerative phenotype. Friedreich ataxia (FRDA) is a relentlessly progressive neurodegenerative disorder caused by GAA·TTC repeat expansion within the first intron of the frataxin (*FXN*) gene [6]. FRDA is autosomal recessive and is the only currently known human disorder associated with GAA·TTC repeat expansion. The normal range of GAA·TTC sequences within this intronic region is between 6 and 36 repeats, while affected individuals have expansions ranging from 120 to 1700 uninterrupted repeats, most commonly 600 to 900 triplets [6]–[9]. Transcription-dependent structure formation by expanded GAA·TTC repeats and/or heterochromatin-mediated gene silencing have been proposed as likely causes of reduced *FXN* expression in FRDA [10]–[16]. The length of the repeat tract directly correlates with disease severity [17],[18], but our current understanding of the mechanisms governing GAA·TTC repeat expansion in FRDA is incomplete and is the focus of this study.

While intronic GAA·TTC sequences within the normal size range (<36 triplets) are stably maintained, uninterrupted premutation (36–120 triplets) and expanded (>120 triplets) alleles display intergenerational and somatic instability, consisting of both contraction and expansion [8], [9], [19]–[27]. Interruptions within the repeat tract stabilize premutation alleles during germline transmission [9] and in peripheral leukocytes from GAA·TTC carriers [24], but the effects of interruptions on the stability of expanded alleles have not been reported. The intergenerational dynamics of GAA·TTC instability are dependent on the mode of inheritance; paternal transmission results in a bias towards repeat contraction, while maternal transmission leads to both repeat expansion and contraction [19],[20],[22]. Somatic instability in FRDA appears to be tissue-specific as to whether repeat contraction or expansion predominates. Analysis of GAA·TTC allele size in multiple tissues from FRDA patients found a general contraction bias during aging in most tissues examined [26]. However, a bias towards age-dependent expansion is seen in disease relevant tissues of FRDA patients, notably the dorsal root ganglia (DRG) of the central nervous system, which suggests that the somatic expansion bias in these tissues directly contributes to disease progression [27].

The unstable nature of the disease-associated repeat sequences is generally attributed to the ability of these sequences to adopt non-B DNA structures [3],[4], but the cellular processes potentiating instability have yet to be fully elucidated. Studies using bacteria, yeast, and patient cell lines demonstrated a strong association between replication and GAA·TTC instability, consisting mostly of contractions [24], [28]–[30]. The somatic expansion bias within post-mitotic neurons of the spinal cord suggests that mechanisms other than replication, such as transcription and/or post-replicative DNA repair, could be the primary forces driving GAA·TTC repeat expansion in FRDA patients. GAA·TTC repeat sequences have been shown to adopt DNA triplex and triplex-associated structures *in vitro* and in bacteria [11]–[14],[31],[32], leading to stalled transcription within the promoter distal half of the GAA·TTC repeat region [13],[14]. The association between transcription and structure formation by GAA·TTC repeat sequences, coupled with the high levels of *FXN* expression in the spinal cord [6], suggests that there may be a relationship between transcription and GAA·TTC expansion. However, the contribution of transcription to GAA·TTC expansion within the genomic context has not been thoroughly examined.

The current lack of information regarding the mechanisms responsible for GAA·TTC expansion in FRDA is partly due to the absence of a good experimental model for GAA·TTC repeat expansion. Bacterial and yeast models of GAA·TTC instability display a pronounced contraction bias [24],[29],[30]. While a recently developed system was able to capture the rare GAA·TTC expansion events in yeast by a selection scheme, this limited analysis to single-event expansions [33]. Lymphoblastoid cell lines derived from FRDA patients have proven to be inconsistent models for the analysis of GAA·TTC instability and require meticulous small-pool PCR techniques to analyze the rare expansion events [23],[34]. While mouse models have proven valuable in reproducing the tissue-specific expansion seen in FRDA patients [35],[36], a more homogeneous and rapid system readily capable of experimental manipulation would provide a valuable tool for mechanistic studies of GAA·TTC repeat expansion.

Here we present a novel model for studying trinucleotide repeat expansion in human cells. In our model system, uninterrupted GAA·TTC repeat sequences display high levels of genomic instability, with an overall tendency towards progressive expansion. Using this system, we characterize the relationship between repeat length and expansion. We further differentiate key mechanistic processes regarding GAA·TTC expansion in human cells. We demonstrate that GAA·TTC repeat expansion in our model is independent of cell division rates. Using unique reporter constructs, we identify transcription through the repeat tract as a major contributor to GAA·TTC repeat expansion.

## Results

### GAA·TTC Repeat Sequences Undergo Progressive Expansion in Human Cell Lines

**Figure 1:**
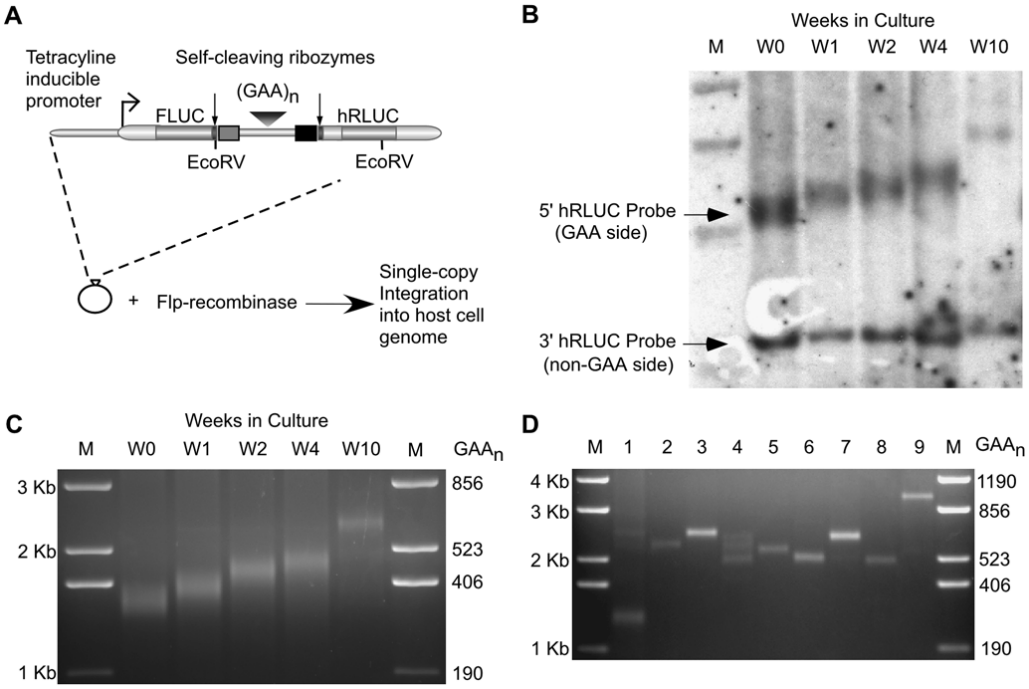
Model of GAA·TTC repeat expansion in human cell lines. (A) Tandem reporter construct designed to isolate transcription elongation through GAA·TTC repeat insert sequences. Single copy integration of the construct into the genome of the host cell line is facilitated via conservative, site-specific recombination using Flp-recombinase. (B) Southern blot analysis of GAA·TTC repeat expansion using the template DNA isolated from a human cell line with a (GAA·TTC)_352_ insert after 0, 1, 2, 4, and 10 weeks (W0–W10) in culture. M: 1 Kb plus size standard. EcoRV digestion of the genomic DNA cuts the tandem construct upstream of the GAA·TTC repeat region and between the 5′ hRLUC and 3′ hRLUC regions. 5′ hRLUC probe is specific to the 5′ side of the hRLUC reporter in the tandem construct containing the GAA·TTC insert region. 3′ hRLUC probe is specific to the 3′ region of the hRLUC expression cassette in the tandem construct and is not associated with the GAA·TTC repeat region. (C) PCR analysis of a (GAA·TTC)_352_ repeat insert isolated at W0, W1, W2, W4, and W10 from Figure 1B. PCR amplification adds 438 bp to the GAA·TTC insert (5′: 338 bp+(GAA)_n_+100 bp: 3′). M: 1 Kb plus size standard (D) PCR analysis of GAA·TTC repeat inserts from clonal cell isolates derived from an end-point dilution of the (GAA·TTC)_352_ parental cell line at W4 in Figure 1B and 1C. PCR amplification adds 438 bp to the GAA·TTC insert. M: 1 Kb plus size standard.

In order to analyze the dynamics of GAA·TTC repeat stability within the context of the human genome, we utilized tandem reporter constructs containing uninterrupted (GAA·TTC)_n_ repeat arrays integrated into the genome of an HEK 293 host cell line (Figure 1A). The tandem reporter constructs utilize two self-cleaving ribozymes in order to isolate transcription elongation through the insert region of the construct. These constructs have been previously tested and characterized [37]. Stable cell lines were established using Flp-recombinase mediated recombination, which allowed for single-copy integration at a consistent chromosomal location and orientation in all cell lines used in this study. Antibiotic selection following construct transfection produced colonies derived from individual cells with an integrated reporter construct (see Materials and Methods). Therefore, all cell lines used in this study are single-cell clonal lineages. We confirmed single-copy integration by Southern blot analysis (as shown in Figure 1B). We constructed the (GAA·TTC)_n_ repeat arrays using an *in vitro* ligation strategy [38] in order to circumvent bacterial propagation, which often leads to the contraction of large GAA·TTC sequences and could result in the preferential selection of repeats stabilized by interruptions. The repeat inserts were sequenced prior to transfection to ensure that the repeat tracts in our cell lines were uninterrupted.

We analyzed the stability of GAA·TTC repeat sequences within our cell lines (Figure 1B and 1C). A (GAA·TTC)_352_ insert was initially chosen in order to analyze the stability of a repeat allele within the size range expected to be unstable in FRDA. A cell line made with a (GAA·TTC)_352_ insert was serially passaged over a 10 week period. Genomic DNA samples were isolated at Day 0 (W0) and after 1, 2, 4, and 10 weeks in culture (W1–W10). Subsequent sizing of the GAA·TTC insert by Southern blot (Figure 1B) and PCR (Figure 1C) analyses showed the progressive expansion of the GAA·TTC repeat insert as a function of time in culture. Expansion was detected as early as W1 (Figure 1B and 1C). Southern blot analysis confirmed that the observed instability is restricted to the (GAA·TTC)_n_ region of the integrated construct (Figure 1B). EcoRV digestion of the genomic DNA cuts the integrated tandem construct upstream of the GAA·TTC insert region and between the 5′ hRLUC and 3′ hRLUC region of the construct (Figure 1A). Our results show that the expansion is localized to the 5′ hRLUC probe region containing the GAA·TTC insert, while the 3′ hRLUC probe region remains stable (Figure 1B). PCR analysis of the GAA·TTC insert reproduced the results obtained by the Southern blot analysis and showed a gain of roughly 119 triplets at W4 (Figure 1C) and a gain of 284 triplets at W10 (Figure 1C). PCR and sequencing analysis of the DNA sequence flanking the GAA·TTC repeat region showed that instability is restricted to the repeat tract, while the flanking sequence remains unaffected (Figure S1 and Figure S2). PCR analysis allows for a more efficient and accurate analysis for GAA·TTC insert sizing and will be used for sizing analysis throughout this study.

We wanted to further characterize the dynamics of GAA·TTC instability within the cell population during culturing by analyzing the size distribution of individual GAA·TTC repeat alleles in that population. End-point dilution of the parental (GAA·TTC)_352_ cell line at W4 (Figure 1B and 1C) produced colonies derived from individual cells within the population. Size analysis of GAA·TTC sequences from these clonal cell isolates revealed a mixed pool of GAA·TTC repeat alleles, ranging in size from 264 to 1000 repeat units (Figure 1D). Of 9 clones, 8 represented expansion events relative to the transfected (GAA·TTC)_352_ insert and one deletion product was detected (lane 1 in Figure 1D). In several of these colonies, multiple amplification products were detected. The detection of different sized alleles in the individual colonies is likely due to continued instability during the growth of the colony. The mixed distribution of repeat alleles in Figure 1D illustrates the mosaicism of individual GAA·TTC repeat alleles within the cell population, which is a characteristic commonly seen in the somatic tissues of FRDA patients [26],[27]. The wide-ranging instability of individual repeat alleles illustrated in Figure 1D suggests that the progressive expansion displayed in Figure 1B and 1C does not represent the uniform expansion of every allele in the cell population. Figure 1B and 1C likely represent an expansion bias among the majority of repeat alleles, with larger and smaller outlier alleles within the population. PCR and Southern blot analysis of a large pool of mixed sized repeat alleles is prone to detect the most common alleles in that population, therefore the products shown in Figure 1B and 1C are likely a reflection of this tendency. It is important to note that there is no selective pressure acting on the GAA·TTC repeat inserts within the integrated reporter construct during culturing. Therefore, there is unlikely to be any sampling bias favoring repeat expansion over deletion.

### GAA·TTC Expansion Is Repeat Length–Dependent

**Figure 2:**
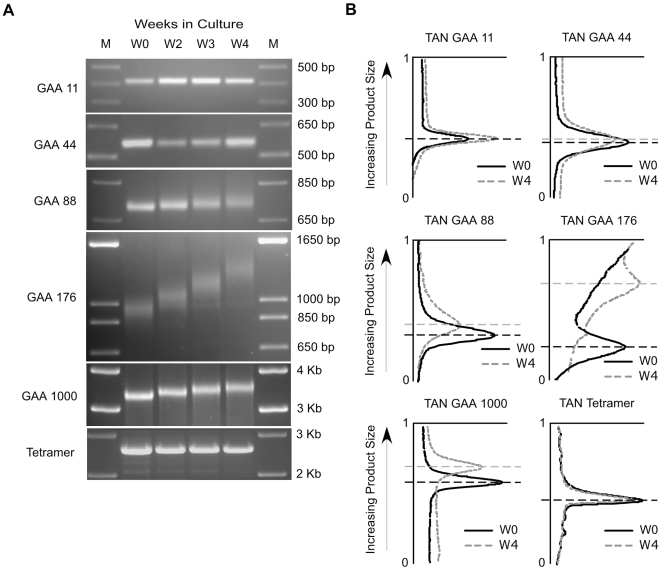
Expansion is specific to GAA·TTC repeats and is repeat length–dependent. (A) PCR analysis of (GAA·TTC)_11_, (GAA·TTC)_44_, (GAA·TTC)_88_, (GAA·TTC)_176_, and (GAA·TTC)_1000_ inserts isolated at W0, W2, W3, and W4. The tetramer insert sequence is a 2.1 Kb semi-repetitive sequence controlling for the effects insert size on stability. PCR amplification adds 438 bp to the GAA·TTC and tetramer inserts. M: 1 Kb plus size standard. (B) Profile analysis of the insert PCR product mobility distribution. Profile analysis of inserts isolated at W0 and W4 are shown. The x-axis represents product signal intensity. The y-axis represents product mobility during electrophoresis. Dashed lines mark the mobility of the peak amplification signals at W0 and W4. Profile analysis was performed using Kodak Molecular Imaging software.

We next analyzed the relationship between repeat length and stability within our cell lines (Figure 2). The duration between construct transfection and initial insert sizing varies among the different clones (see Materials and Methods). The repeat lengths used throughout this report are in reference to the transfected insert sequence and do not account for any gains in repeat size between transfection and the beginning of the time-course experiments. Time-course analysis of repeat stability was performed using cell lines harboring 11, 44, 88, 176, and 1000 GAA·TTC repeats (Figure 2A). (GAA·TTC)_11_ inserts are within the normal size range of GAA·TTC repeats found within the first intron of the *FXN* allele and repeats within this range have been shown to be below the reported initiation threshold for instability in simple replication models and human cells [23],[24],[38]. As expected, the (GAA·TTC)_11_ insert sequence remained stable over the 4 week time-course as indicated by the tight banding pattern of the PCR amplification product at each time-point (Figure 2A). The (GAA·TTC)_44_ underwent a modest level of expansion, which is in agreement with the initiation threshold for instability reported by others [23],[24] (Figure 2A). PCR mobility profile analysis (Figure 2B) further illustrates the stability of the (GAA·TTC)_11_ insert and the modest expansion of the (GAA·TTC)_44_ repeat insert during the time-course experiments. Substantial expansion of the (GAA·TTC)_88_ and (GAA·TTC)_176_ inserts was observed during the 4 week time-course with the larger (GAA·TTC)_176_ sequence demonstrating a more rapid expansion when compared to the (GAA·TTC)_88_ sequence (Figure 2A). PCR mobility profile analysis of the (GAA·TTC)_88_ sequence showed a gain of 9 triplets when comparing the peak intensities of the amplified products at W0 and W4, while the (GAA·TTC)_176_ sequence showed a gain of 140 triplets over the same time period (Figure 2B). These results demonstrate that GAA·TTC repeat sequences undergo a length-dependent increase in expansion rate in our cellular model.

Previous analysis using peripheral blood samples of FRDA patients revealed a contraction bias in GAA·TTC sequences greater than 500 repeats in length [23], while post-mortem analysis of GAA·TTC sequences ranging from 350–1030 repeats in the dorsal root ganglia of FRDA patients demonstrated an age-dependent expansion bias [27]. To analyze the stability dynamics of larger GAA·TTC sequences within our cell lines, we performed time-course experiments using a cell line isolated from the end-point dilution of the (GAA·TTC)_352_ cell line (lane 9 in Figure 1D). This cell line contained a (GAA·TTC)_1000_ insert sequence (Figure 2A). Sizing of the insert sequence showed the continued expansion of the (GAA·TTC)_1000_ insert from W0 to W4 (Figure 2A and 2B), confirming that larger GAA·TTC inserts continue to expand in our system.

To determine if the observed expansion is due to the length of the DNA sequence inserted into poly-linker region of our construct, rather than sequence composition, we analyzed the stability of a semi-repetitive 2.1 kilobase (kb) tetramer insert sequence (Figure 2A). The tetramer sequence is composed of four identical non-repetitive 529 bp fragments and is equivalent in size to a (GAA·TTC)_700_ insert sequence [37]. We have previously shown that this tetramer insert has a neutral affect on transcription through the insert region of the reporter constructs [37]. The tetramer sequence remained stable over the duration of the time-course (Figure 2A and 2B), indicating that the observed expansion of GAA·TTC repeat sequences in these cell lines is not solely a function of insert length. This suggests that GAA·TTC expansion in our cell lines must be due to certain properties intrinsic to these triplet repeats.

### Repeat Interruptions Reduce GAA·TTC Expansion Levels

**Figure 3:**
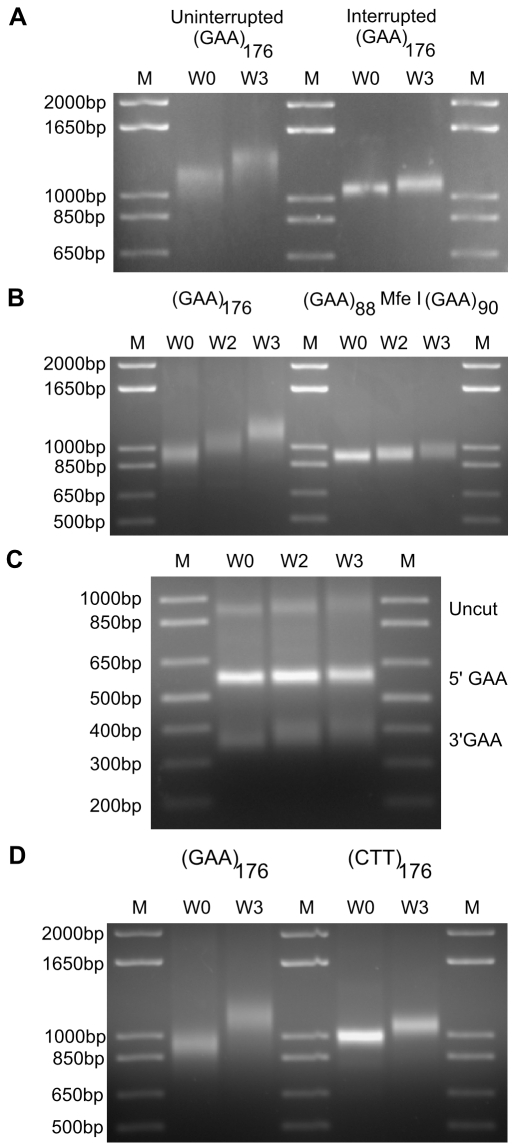
Expansion levels are affected by the purity of the repeat sequence and the repeat orientation. (A) PCR analysis of GAA·TTC expansion within an uninterrupted (GAA·TTC)_176_ insert at W0 and W3 compared to a (GAA·TTC)_176_ insert with two interrupting mutations identified by sequencing analysis. An A→T mutation was detected approximately 118 triplets into the repeat region from the 5′ end and a T→G mutation was detected 31 triplets into the repeat region from the 3′ end. PCR amplification adds 438 bp to the GAA·TTC insert. M: 1 Kb plus size standard. (B) PCR analysis of GAA·TTC expansion within an uninterrupted (GAA·TTC)_176_ insert at W0, W2, and W3 compared to a (GAA·TTC)_88_MfeI(GAA·TTC)_90_ insert sequence containing an interrupting hexamer sequence located between two repeat tracts. The CAATTG hexamer is the recognition sequence for the Mfe I restriction endonuclease. PCR amplification adds 438 bp to the GAA·TTC insert. M: 1 Kb plus size standard. (C) Mfe I digestion of the PCR amplification products from the (GAA·TTC)_88_MfeI(GAA·TTC)_90_ time-course at W0, W2, and W3. PCR amplification adds 338 bp to the 5′ end of the GAA·TTC insert and 100 bp to the 3′ end. Mfe I digestion yields two distinct fragments representing the promoter proximal (GAA·TTC)_88_ repeat tract (5′ GAA) and the distal (GAA·TTC)_90_ repeat tract (3′ GAA). The residual full-length product (Uncut) is due to incomplete digestion. M: 1 Kb plus size standard. (D) PCR sizing analysis of a (GAA·TTC)_176_ insert at W0 and W3 compared to a reverse oriented (CTT·AAG)_176_ insert sequence at the same time-points. PCR amplification adds 438 bp to the repeat insert. M: 1 Kb plus size standard. A representative gel from an *n* = 2 is shown.

Previous studies have shown that interruptions in the purity of the GAA·TTC repeat tract have a stabilizing effect on these sequences [9],[24], possibly by interfering with the formation of secondary structures by the repeat. Time-course analysis of repeat stability was performed on four individual cell lines with (GAA·TTC)_176_ insert sequences. The mean increase in repeat size among three of these cell lines was 61.1±11.6 triplets, while the fourth cell line gained only 12 triplets after 3 weeks in culture (Figure 3A). Sequencing of the first three inserts did not detect any interruptions. Sequencing of the GAA·TTC insert region within the fourth cell line identified two separate interrupting point mutations (Figure S3). An A→T mutation was detected approximately 118 triplets into the repeat region from the 5′ end and a T→G mutation was detected 31 triplets into the repeat region from the 3′ end (Figure S3). Expansion of the repeat sequence either upstream or downstream of the interruption distributes the base signal when sequencing from a polymorphic population, as represented in Figure S3. These shifts can mask the detection of potential interruptions during automated sequencing, with mutations towards the edges of a repetitive run being more readily detected. Careful visual inspection of the primary sequencing data is needed when seeking to identify potential interruptions within unstable tandem repeat arrays. The identification of multiple interruptions within the GAA·TTC sequence in this cell line suggests that even a couple of point mutations that interrupt the purity of the repeat sequence can greatly reduce the rate of repeat expansion.

To further analyze the effects of interruptions on GAA·TTC repeat expansion, we created a cell line containing a repeat insert with an interrupting hexamer (TCAATT) that creates an Mfe I restriction endonuclease recognition site situated between two repetitive tracts, (GAA)_88_MfeI(GAA)_90_. Sequencing did not detect any interruptions in the either of the two repeat tracts flanking the interruption within this construct, but visual inspection of the sequencing chromatogram confirmed the presence of the interrupting hexamer (Figure S3). Stability analysis revealed that this interrupting hexamer sequence reduces the rate of expansion when compared to uninterrupted (GAA·TTC)_176_ insert sequences (Figure 3B). The (GAA)_88_MfeI(GAA)_90_ insert gained only 22 triplets after 3 weeks in culture, compared to the 61.1±11.6 mean triplet gain by the uninterrupted (GAA·TTC)_176_ insert sequences. The primer pair used for PCR amplification adds 338 bp to the 5′ end of the GAA·TTC insert and 100 bp to the 3′ end. Digestion of the amplified product containing the Mfe I site interruption yields two distinct bands representing the two repeat tracts, which allowed us to investigate whether there is preferential expansion at one end of the repeat or expansion from both sides of the interrupting mutation (Figure 3C). Analysis of the digested products at W0, W2, and W3 showed more expansion within the promoter distal (GAA·TTC)_90_ repeat tract than within the promoter proximal (GAA·TTC)_88_ repeat tract (Figure 3C). Incomplete digestion was used to highlight the visible expansion of the full-length fragment, which indicates that the observed preferential expansion within the promoter distal tract is not simply due to differential mobility between the two digested fragments. Complete digestion at each time-point has been achieved at lower DNA concentrations (not shown), thereby excluding the possibility that a portion of the interrupting mutations were lost during culturing. These results confirm that GAA·TTC expansion rates are affected by the purity of the repeat sequence and suggest that expansion is biased towards the promoter distal end of the repeat tract.

### GAA·TTC Expansion Levels Are Affected by Repeat Orientation

The stability of GAA·TTC repeat sequences is affected by the orientation of the repeat array during plasmid vector propagation in bacteria, yeast and transiently transfected mammalian cells [24], [39]–[42]. To analyze the effects of repeat orientation on GAA·TTC expansion in our system, we created cell lines with reverse oriented (CTT·AAG)_176_ insert sequences in our expression constructs. The rates of expansion within these reverse oriented inserts were compared to the expansion rates of the forward oriented insert sequences (Figure 3D). Two separate reverse oriented (CTT·AAG)_176_ inserts gained 33 and 26 triplets, compared to the 61.1±11.6 mean triplet gain by the forward oriented (GAA·TTC)_176_ inserts after 3 weeks in culture. Sequencing did not detect any interruptions in the purity of either reverse oriented insert. These results demonstrate that GAA·TTC expansion levels are affected by the orientation of the repeat sequence within the genome.

### GAA·TTC Expansion Is Independent of Cell Division Rates

**Figure 4:**
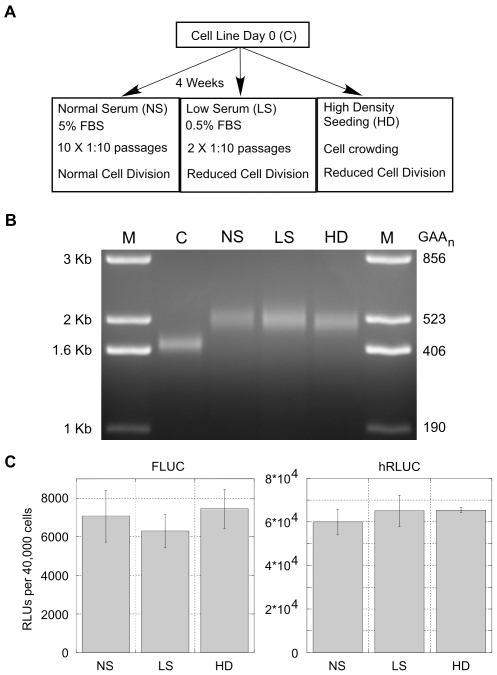
GAA·TTC expansion is independent of cell division rates. (A) To analyze the influence of cell division on GAA·TTC expansion, a parental cell line was cultured under various conditions affecting cell-division rates: Normal Serum (NS, 5% FBS growth media) in which cells were passaged 10 times over the 4 week time period; Low Serum (LS, 0.5% FBS growth media) in which cells were passaged only twice over the 4 week time period due to reduced cell division; High Density Seeding (HD) cells were plated and carried near confluent cell levels in normal growth media in order to reduce cell division due to crowding. (B) PCR analysis of a (GAA·TTC)_352_ insert from a cell line cultured for 4 weeks in various conditions affecting cell-division rates. C is the Day 0 control. PCR amplification adds 438 bp to the GAA·TTC insert. M: 1 Kb plus size standard. A representative gel from an *n* = 3 is shown. (C) Analysis of the effects of the different growth conditions on transcription levels through the integrated reporter constructs. Analysis of the FLUC and hRLUC reporter expression levels is shown. Expression is represented as relative light units (RLUs) per 40,000 cells. Error bars represent the standard error of the mean (SEM) from an *n* = 3.

Replication has been shown to influence GAA·TTC repeat stability in previous model systems [24], [28]–[30],[39]. To analyze the influence of replication on GAA·TTC expansion in our cell lines, we sought to alter cell division rates during culturing and analyze the influence on GAA·TTC stability (Figure 4). A cell line containing a (GAA·TTC)_352_ insert was cultured in low-serum growth media (LS, 0.5% FBS), which resulted in a 5–10 fold decrease in cell division rate. During the 4 week experimental period, cell lines grown in LS media were passaged only twice due to reduced cell division, while cell lines carried in normal growth media (NS, 5% FBS) underwent 10 passages. Expansion rate of the (GAA·TTC)_352_ insert was unaffected after 4 weeks in LS growth media when compared to the control insert grown in NS growth media for 4 weeks (Figure 4B). An additional time-course was performed in which the cell lines were grown in normal growth media (5% FBS) at or near confluency in order to reduce cell divisions due to crowding (HD in Figure 4). The cell lines were split and reseeded at ∼90% confluence every third day. Sizing analysis demonstrated that the expansion rate of the (GAA·TTC)_352_ insert was unaffected by cellular confluency when compared to the control NS insert (Figure 4B). Luciferase reporter expression levels showed that basal transcription levels within the integrated reporter constructs remain unchanged during culturing in the various growth conditions (Figure 4C). These results demonstrate that the observed GAA·TTC expansion is independent of replication rate or cell confluency in our cellular model and further indicates that GAA·TTC repeat expansion in our model correlates better with time in culture than with the number of cell divisions.

### Decreased Transcription Levels Reduce GAA·TTC Expansion Rates

Induced transcription levels have previously been shown to promote the contraction of CAG·CTG and GAA·TTC repeat sequences in human cell lines [43]–[45]. We found a similar response in our model system (Figure S4). While these previous studies aimed to produce instability through induced transcription from stable alleles, this current study differs in that the GAA·TTC repeat sequences undergo robust expansion at basal transcription levels. The human cytomegalovirus immediate-early enhancer/promoter (CMVIE) is well known for directing very high levels of transgene expression in human cells. In our construct, this promoter is regulated by a pair of tetracycline operator sites near the transcription start site. While this affords a degree of inducibility, luciferase expression values in our cell lines indicated high basal levels of transcription through our constructs in the absence of promoter induction (Figure 4C). Background transcription is likely due to promoter leakage, but could be due to high levels of local transcription within the genomic region near our constructs.

**Figure 5:**
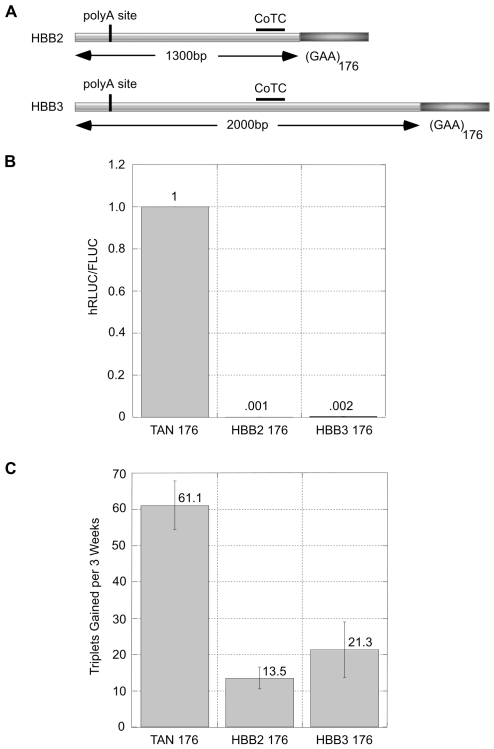
Decreased transcription levels reduce the rate of GAA·TTC expansion. (A) Modified tandem reporter constructs containing segments of the human β-globin (*HBB*) transcription termination sequence inserted upstream of the (GAA·TTC)_n_ insert region. HBB2 is a 1,300 bp fragment containing the poly(A) addition site of *HBB* and the cotranscriptional cleavage (CoTC) element. HBB3 is a 2,000 bp fragment containing the poly(A) site, CoTC element and additional downstream sequence. (B) Analysis of transcription rates through the GAA·TTC repeat insert sequences in the TAN, HBB2, and HBB3 (GAA·TTC)_176_ constructs. Successful transcription through the repeat inserts is expressed as the ratio of hRLUC/FLUC luciferase reporters located in the tandem constructs. Values are normalized to the TAN control construct. The error bars indicate the SEM for an *n* = 3. (C) Analysis of GAA·TTC expansion within the TAN, HBB2, and HBB3 constructs after 3 weeks in culture. (GAA·TTC)_176_ inserts were analyzed by PCR amplification at W0 and W3 and the number of triplets gained during this 3-week period is shown. Experiments were performed using three separate cell lines for each construct. The mean gain in triplets is shown for an *n* = 3. Errors bars represent the SEM for *n* = 3; p<.05 for both HBB2 and HBB3 when compared to the control TAN construct.

Since expansion occurs in the absence of promoter induction, we wanted to further reduce as much as possible any transcription through the repeat region of our construct by utilizing the well characterized transcription termination signals of the human β-globin gene (*HBB*; NM_000518). We have previously created tandem constructs designed to test the efficiency of transcription termination by defined sections of the polyadenylation sequence from *HBB* [37]. Here, we introduced these transcription termination sequences upstream of the GAA·TTC inserts within the polylinker region of the tandem construct in order to reduce transcription through the repeat tract (Figure 5A). The HBB2 sequence is a 1300 bp segment containing the poly(A) addition site and the putative co-transcriptional cleavage (CoTC) element within *HBB*, which is thought to enhance transcription termination [46]–[48]. The HBB3 sequence is a 2000 bp segment also containing the poly(A) site and CoTC element plus additional downstream sequence. By analyzing the expression ratio of the hRLUC and FLUC reporters in our constructs, we were able to quantitate transcription rates through the GAA·TTC insert region (Figure 5B). The HBB2 and HBB3 sequences reduce transcription through the repeat region to less than 1% (.001 and .002 respectively) of the control TAN construct (Figure 5B), which is in agreement with our previous findings [37].

To analyze the effects of decreased transcription on GAA·TTC expansion over time, we performed time-course analyses of repeat stability in the TAN, HBB2, and HBB3 constructs with (GAA·TTC)_176_ insert sequences (Figure 5C). A significant (p<.05 for HBB2 and HBB3) decrease in expansion levels was observed when the GAA·TTC repeat sequences were positioned downstream of the transcription termination elements in HBB2 and HBB3 as compared to the control TAN construct (Figure 5C). After 3 weeks in culture, the (GAA·TTC)_176_ inserts gained 13.5±5.2 triplets when downstream of the HBB2 termination sequence and 21.3±13.4 triplets when downstream of the HBB3 termination sequence. The (GAA·TTC)_176_ inserts gained 61.1±11.6 triplets in the TAN construct (Figure 5C). Sequencing did not detect any interruptions within the GAA·TTC region of the HBB or TAN constructs. The decreased expansion levels in the HBB2 and HBB3 constructs are unlikely to be due to the additional sequence inserted immediately upstream of the GAA·TTC insert region. The HBB3 construct has 700 bp of added downstream sequence that is not present in the HBB2 construct (Figure 5A), therefore the sequence immediately upstream of the GAA·TTC repeat region differs between the two constructs. Analysis of GAA·TTC expansion in a separate construct containing a shorter HBB termination element also reduced expansion levels (Figure S5), but we were only able to obtain a single clone due to the difficulty of creating these cell lines using *in vitro* ligation. These findings indicate that the observed decreased expansion levels within the HBB constructs are not due to the sequence composition inserted upstream of the repeat insert. Although the effects of spacing, or other small differences conferred by the HBB insertions, cannot be ruled out, our results, taken together, indicate that transcription through the repeat tract is a major contributor to expansion. Ideally, we would like analyze the influence of induced transcription on the reduced GAA·TTC expansion levels within the HBB constructs, but transcription termination within these constructs is highly efficient and we do not observe appreciable levels of transcription induction through the repeat region, as analyzed by hRLUC expression levels (data not shown). Our finding that decreased transcription levels reduce GAA·TTC repeat expansion rates supports our hypothesis that transcription contributes to the progressive GAA·TTC repeat expansion seen in human cells. The residual GAA·TTC expansion in the HBB constructs could be due to antisense transcription or to other factors, in addition to transcription, that contribute to GAA·TTC repeat expansion.

## Discussion

In this study, we present a human cellular model of progressive GAA·TTC repeat expansion. Our model recapitulates key features of GAA·TTC instability in FRDA. The continued expansion of large GAA·TTC repeats and the observed mosaicism of repeat alleles in our system are characteristic of instability within the dorsal root ganglia (DRG) of FRDA patients [27], one of the primary affected tissues in FRDA. The level of instability and prominent expansion bias in our system has not been achieved in previous cellular models of trinucleotide repeat instability. Examination of GAA·TTC instability in patient samples requires the analysis of thousands of individual repeat alleles using small-pool PCR in order to detect significant levels of variability in the cell population [26],[27]. In our cellular model, we are able to detect high levels of expansion in a matter of weeks using standard PCR techniques. The robust expansion observed in our system could be aided by the lack of selective pressure against repeat expansion in our luciferase reporters. In FRDA patient cells, the continued expansion of GAA·TTC repeats within the first intron of the *FXN* gene results in the length-dependent reduction of frataxin expression, which eventually leads to cell death. Larger repeat alleles would be selected against as the dying cells are removed from the population, resulting in larger alleles going undetected during PCR sizing analysis. Tissues samples from FRDA patients and mouse models used for GAA·TTC repeat analyses are likely to be heterogeneous mixtures of different cell types. Any expansion biases specific to certain cell lineages, notably the DRG, would be underrepresented due to the presence of alleles from other cell types in the population. The obvious expansion bias observed in our model system could be due to the homogenous nature of the cells in culture. The continuous expansion of repeat alleles in our model provides us with an advantageous system for mechanistic studies of repeat expansion in human cells.

Errors during mitotic replication, due to structure formation by the GAA·TTC repeat sequence and/or strand-slippage events, were previously proposed as the primary mechanisms by which GAA·TTC repeat expansion occurs [30],[33],[41]. However, replication models of GAA·TTC expansion, derived from data using simple replication systems displaying pronounced contraction biases, generally conflict with instability data obtained from patient studies and mouse models. In FRDA patients and transgenic mouse models, GAA·TTC instability in proliferating tissues consisted predominantly of contraction events [26],[35], while a GAA·TTC expansion bias was predominantly localized to post-mitotic neurons within the spinal cord [27],[36]. While replication may promote GAA·TTC contraction, we hypothesize that GAA·TTC expansion occurs via a separate non-replicative mechanism. Given that it is repeat expansion that results in disease, it is important to distinguish repeat contraction from repeat expansion during mechanistic studies of trinucleotide repeat instability.

We have shown that GAA·TTC expansion in our cell lines is independent of cell division rates, which supports our hypothesis that GAA·TTC expansion, unlike contraction, is not mechanistically linked to cellular replication. Previous reports using plasmid replication models in *E. coli*, *S. cerevisiae* and transiently transfected mammalian cells demonstrated a relationship between repeat stability, repeat orientation, and repeat distance from replication origins [28],[30],[39],[41]. A common finding among these studies was that GAA·TTC repeats demonstrate higher levels of instability, consisting mostly of contractions, when the purine (GAA) strand serves as the template for lagging strand synthesis. Recently, Shishkin *et al.* [33] found that GAA·TTC expansion in yeast was unaffected by the repeat orientation relative to replication origins and that, unlike repeat contraction, replication fork stalling was not involved in GAA·TTC expansion. In our model, reversing the repeat orientation decreases the rate of expansion, yet we have shown that expansion in the less stable forward orientation is independent of cell division. Therefore the differential stability between the two orientations in our system is unlikely to involve replication fork dynamics. While we are unable to rule out possible chromosomal positioning effects on GAA·TTC stability in our system, the strong expansion bias and the relatively low level of repeat contraction could be due to the lack of replication-mediated instability in our cellular model.

Using novel tandem reporter constructs, we have shown that transcription levels through the repeat tracts contribute to GAA·TTC expansion in our model system. By introducing the polyadenylation signal and terminator of the *HBB* gene upstream of the repeat, and thereby reducing transcription through the repeat tract, we were able to decrease expansion levels. This is the first report to establish a relationship between transcription levels and GAA·TTC expansion rates, and to correlate transcription levels with the expansion of any disease-associated trinucleotide repeat sequence in human cells. Our findings support a transcription-dependent mechanism for GAA·TTC expansion. Transcription-dependent expansion is consistent with GAA·TTC instability data obtained from patient samples and mouse models, which found an expansion bias within the neurons of the dorsal root ganglia [27],[36], where *FXN* expression levels are the among the highest of all tissues [6].

Structure formation by the GAA·TTC repeat sequence is likely to be a key event in the mechanism leading to GAA·TTC expansion. Our group has previously shown that transcription through expanded GAA·TTC repeat sequences is associated with the formation of a transient DNA triplex structure and an RNA·DNA hybrid *in vitro* and in live bacteria, which leads to transcriptional arrest at the promoter distal duplex-triplex junction [13],[14]. Our finding that GAA·TTC repeat expansion is biased towards the distal end of the repeat tract supports a model in which structure formation and stalled transcription complexes at the promoter distal end of the repeat tract facilitates expansion within this region. We have shown that expansion in our cellular model initiates at a length of approximately 44 triplets and this repeat length correlates with the shortest repeat found to be associated with RNA·DNA hybrid formation in our earlier study [14], which suggests that structures, such as a transient RNA·DNA hybrid, are involved in the expansion process. Out of register re-annealing of the non-template strand after removal of the RNA hybrid could result in the formation of slipped-stranded structures, which are thought to be key intermediates in the process leading to CAG·CTG expansion [49]. Interruptions in the purity of the repeats would reduce the likelihood of slipped-strand formation by acting as a reference point for annealing within the repeat tract and would thereby reduce expansion levels, as demonstrated in this study. We have also shown that reversing the orientation of the repeats relative to the promoter reduces expansion levels. This effect could be due to an altered potential for transcription-associated structure formation in the reverse orientation relative to the promoter. While Shishkin *et al.* [33] demonstrated that GAA·TTC expansion was unaffected by the repeat orientation relative to replication origins, the orientation of the repeat relative to the promoter in their constructs remained constant and any orientation-dependent transcriptional influence on expansion could have been overlooked. While we observed reduced expansion levels in the HBB constructs and in the TAN constructs with reverse oriented repeats, expansion was not completely abolished. It is possible that antisense transcription passes through the GAA·TTC insert, which we cannot detect with our reporters. Antisense transcription could be responsible for the residual expansion within these constructs.

Components of the DNA repair machinery have been implicated in the expansion of CAG·CTG repeat sequences [50]–[56], but this association has not yet been made regarding GAA·TTC repeat sequences. Any transcription-mediated model of GAA·TTC expansion is likely to involve post-replicative DNA repair due to the requirement of newly synthesized DNA to facilitate this expansion. In studies utilizing triplex-forming oligonucleotides (TFOs), components of the nucleotide excision repair pathway (NER) were shown to bind DNA triplex structures *in vitro* [57],[58] and triplex-associated mutagenesis was found to be dependent on the transcription-coupled NER (TC-NER) repair pathway in mammalian cells [59]. The mismatch repair (MMR) complex MutSβ (MSH2–MSH3), which is required for CAG·CTG expansion in mice, was also shown to interact with the NER machinery in the recognition of TFO-directed psoralen DNA inter-strand cross-links [60], suggesting that MutSβ may be involved in the recognition and repair of DNA triplex structures. Slipped-strand structures formed by GAA·TTC repeats could also be recognized and processed by MutSβ, as has been shown for CAG·CTG repeats [49],[61]. Therefore, transcription-associated structure formation by the GAA·TTC repeat sequence and the subsequent arrest of transcription could lead to the induction of NER, TC-NER, MMR, or an interaction between these various pathways that would lead to expansion during repair. Strand-slippage and/or reiterative synthesis of repeat units during the gap-filling stage of the excision repair pathways would lead to the recursive accumulation of small expansion events, which would account for the progressive GAA·TTC expansion observed over weeks in our cellular model and in the dorsal root ganglia of FRDA patients during aging.

Interestingly, when transcription was induced within the TAN constructs, we observed a decreased rate of expansion and an increase in deletion products during prolonged periods of culturing. This increase in repeat contraction during periods of induced transcription agrees with earlier studies examining CAG·CTG repeat contraction in human cells [43],[44]. Both in our system and the selection assay for CAG·CTG contraction reported by Lin *et al.* [43] induced transcription is driven by modified CMVIE promoters, which are well known for generating high levels of transgene expression. Chromosome fragile sites are often linked to regions of repetitive DNA, including trinucleotide repeats. GAA·TTC repeats were previously shown to be frequent sites of double-strand breaks in yeast [41]. Very high levels of transcription generated during promoter induction could promote strand breaks within the repeat region, the repair of which may favor trinucleotide repeat contraction over expansion. We believe that the GAA·TTC repeat contraction observed during high transcription levels and the incremental GAA·TTC expansion seen in our system at basal and reduced transcription levels occur via separate mechanisms. Both in bacteria and yeast, double-strand breaks within the GAA·TTC repeat region were shown to promote rapid repeat contraction [41],[62], leading us to propose that the repair of double-strand breaks generated during extended periods of induced transcription is responsible for the observed repeat contraction over time. Basal transcription levels within the TAN construct or the reduced transcription levels within the HBB constructs are likely to be more representative of transcription levels produced from the native *FXN* gene.

This study has identified transcription levels as a key regulator of GAA·TTC expansion in human cells. Progressive GAA·TTC expansion in the neurons of FRDA patients has been postulated to contribute to disease progression during aging. Transcription-driven expansion could partially explain the expansion bias of GAA·TTC repeats in the post-mitotic neurons of FRDA patients. The neurons of the DRG are the primary sites of degeneration in FRDA and are among the tissues in which *FXN* gene expression is the highest [6]. The findings of this study provide support for a model in which high gene expression and low cell turnover would promote the progressive expansion of intronic GAA·TTC repeat sequences, thereby reducing *FXN* mRNA levels, causing cell death and neuronal degeneration. Potential therapies aimed at alleviating the transcriptional deficit at the *FXN* gene in FRDA patients should take into consideration the possibility that elevating transcription levels from the *FXN* promoter could exacerbate expansion and inhibit therapeutic effectiveness.

## Materials and Methods

### Construction of Plasmids and Vectors

Construction of the tandem reporter constructs, tetramer insert sequence and polyadenylation regions has been described previously [37]. The capped *in vitro* ligation strategy used to create the (GAA·TTC)_n_ repeat inserts has previously been described [38]. The repeat inserts were cut with SpeI & BamHI and ligated into the tandem vector polylinker region cut with NheI and BamHI. The reverse orientation (CTT·AAG)_176_ inserts were cut with XbaI & BglII and ligated into the tandem vector polylinker region cut with NheI and BamHI. The TCAATT hexamer used to create the Mfe I recognition sequence was added to the 3′ end of a (GAA·TTC)_88_ insert using an oligonucleotide fragment and was ligated to a second (GAA·TTC)_88_ to create the (GAA)_88_T CAATT(G AA)_90_ insert fragment. The non-human sequences (GFP/CAT) flanking the polylinker site serve as unique priming sites for insert sizing. The insert region was sequenced for impurities within the repeat arrays prior to transfection.

### Generation and Maintenance of Cell Lines

Our reporter constructs were integrated into the genome of Flp-In T-REx-293 cell lines (Invitrogen) using the Invitrogen Flp-In T-REx system following the supplier's protocol. Transfection was performed using the Lipofectamine 2000 transfection reagent (Invitrogen). Selection for successful construct integration was performed by culturing transfected cells in media containing hygromycin B (75 µg/ml) and blasticidin-HCL (15 µg/ml). Individual colonies were isolated after approximately 2 weeks under antibiotic selection. The colonies were then expanded for approximately 2 weeks. Insert sizing using PCR analysis was done approximately 4 weeks post-transfection. Cell lines were maintained in Dulbecco's modified Eagle's medium high glucose (Invitrogen) and 5% fetal bovine serum (Sigma) at 5% CO_2_. Time-course experiments were conducted by serially passaging the cell lines. Cells were split 1∶10 approximately every third day using trypsinization. Genomic DNA was isolated at the indicated time-points when the cells were ∼80% confluent. The end-point dilution was performed by seeding the parental cell line at 10–20 cells per 100 mm plate. These cells formed individual colonies that were then isolated and expanded. The genomic DNA was extracted and the GAA·TTC insert was sized.

### PCR Sizing Analysis of GAA·TTC Repeats

Genomic DNA was isolated from the cell lines using DNAzol Reagent (Invitrogen) following the supplier's directions. PCR amplification was performed in 50 µl reactions (100 ng template; 0.2 µM primers; 1 mM dNTP mix (Stratagene); 2.5 units polymerase; 1× enzyme buffer) for 30 cycles. Either Paq5000 DNA polymerase (Stratagene) or Herculase II Fusion polymerase (Stratagene) enzymes were used for PCR amplification using the manufacturer supplied reaction buffer specific to each enzyme. 1.3 M betaine was included in reactions performed using the Herculase II Fusion Enzyme. Primer pairs specific to the TAN construct include: MGF3102 5′-ggtcttgtagttgccgtcgt-3′ forward, MGR3533 5′-caactgactgaaatgcctcaa-3′ reverse; annealing: 58°C; product size: ((GAA)_n_×3)+438 bp. Repeats amplified from the HBB2 constructs were amplified using H2-2574 forward primer: 5′-aggtctgctggctcccttat-3′ with MGR3533 reverse; annealing 55°C; product size: ((GAA)_n_×3)+445 bp. Repeats amplified from the HBB3 constructs were amplified using H3-791 forward primer: 5′- cacagatgattcaataacaaacaaaa-3′ with MGR3533 reverse; annealing 55°C; product size: ((GAA)_n_×3)+501 bp. Amplified products containing 88 GAA·TTC repeat inserts or less were analyzed by electrophoresis using 1.4% agarose gels, while larger fragments were resolved using 1% agarose gels. 1 Kb Plus DNA Ladder (Invitrogen) was used as the size marker. Gels were stained using 1.3 µg/ml ethidium bromide. Gel images were obtained using the Kodak Gel Logic 440 imaging system. Software analysis of the gel images and profile analysis of the insert PCR mobility distribution were obtained using the Kodak molecular imaging software (version 4.0). Graphical representation of the software analysis was created using Prism 4 graphing software and Canvas 8 graphics software. Statistical analysis of GAA·TTC repeat expansion was performed using the Student's t-test for unpaired data with unequal variance.

### Southern Blot Analysis of GAA·TTC Repeat Instability

10 µg of genomic DNA was digested to completion using EcoRV and BglII restriction endonucleases, ethanol precipitated, and resuspended in 1× TE buffer (10 mM Tris, 1 mM EDTA, pH 8). 5× loading buffer (glycerol, 2.5 mM EDTA, bromophenol blue, xylene cyanol) was added to sample(s) before electrophoresis using a 1% agarose gel. The agarose gels containing the digested samples were soaked in 0.1 N HCl for 20 min to depurinate the DNA prior to transfer. The gels were rinsed in distilled water and soaked in 0.4 N NaOH for 15 min to denature the DNA for probe binding. The gels were rinsed in distilled water and set up for transfer. Capillary blot transfer was performed for 12 h using 5× SSC transfer buffer (750 mM sodium chloride, 75 mM sodium citrate) and Hybond-N+ transfer membrane (Amersham Biosciences). Post-transfer, the DNA samples were crosslinked to the membrane using a 1 min UV exposure. [^32^P] Riboprobe synthesis was performed using the pSP72/5′hRLUC (5′ hRLUC probe) and the pSP72/3′hRLUC (3′ hRLUC probe) HindIII linearized templates. Hybridization was performed overnight at 65°C in ULTRAhyb hybridization buffer (Ambion). The hybridized blot was washed in a series of SSC/SDS mixtures increasing in stringency every 5 min. The washed blot was exposed to film for 24 h and 1 week at −80°C.

### Dual Luciferase Assay

The Dual-Luciferase reporter assay system (Promega) was used according to the manufacturers directions. Cells were seeded in a 48-well tissue culture treated plate and induced with doxycycline (1 µg/ml) for 24 h. Cells were washed in PBS and lysed in passive lysis buffer (Promega). Cell lysates were aliquoted into a Greiner 96-well plate (Sigma) and analyzed using a Turner Biosystems Veritas plate-reader luminometer (Turner Biosystems, Sunnyvale, CA) with an integration time of 10 sec according to the Promega dual luciferase reagent protocol. For the culturing experiment, the cells were isolated after 2 weeks in the various culturing conditions and analyzed for luciferase expression. Statistical analysis of relative luciferase expression values was performed using the Student's t-test for unpaired data with unequal variance.

## Supporting Information

Figure S1 PCR analysis of GAA·TTC insert region and flanking sequence stability. PCR amplification was performed on the genomic samples used in Figure 1 at W0 and W10. Primer pairs were designed to amplify separate regions of the integrated tandem reporter construct. Pair 1 amplifies from the 5′ FLUC region to the 5′ hRLUC region (5′: 2347 bp+(GAA)_n_+668 bp: 3′). Pair 2 amplifies the 5′ region flanking the GAA·TTC insert (2272 bp sequence beginning 76 bp upstream of GAA·TTC insert). Pair 3 amplifies the GAA·TTC insert sequence (5′: 151 bp+(GAA)_n_+76 bp: 3′). Pair 4 amplifies the 3′ region flanking the GAA·TTC insert (485 bp sequence beginning 179 bp downstream of GAA·TTC insert).
(2.63 MB TIF)

Figure S2 Sequencing analysis of the immediate flanking region surrounding the GAA·TTC repeat insert. Sequencing analysis was performed on the genomic samples used in Figure 1 at W0 and W10. (A) Sequencing alignment of the 5′ flanking region at W0 and W10. (B) Sequencing alignment of the 3′ flanking region at W0 and W10. The locations of the primers used for PCR analysis in Figure 1 (MGF3102 and MGR3533) and the junctions between the flanking region and the repeat insert are shown.
(5.73 MB TIF)

Figure S3 Sequencing data of GAA·TTC repeat region with interrupting mutations. (A) GAA·TTC repeat region from a clone with 2 point mutations within the repeat region. The left panel is from the 5′ - GAA orientation showing an A→T mutation 118 triplets into the repeat region. The right panel is from the 5′ CTT orientation showing an T→G mutation 31 triplets into the repeat region. (B) GAA·TTC region from a clone in which a TCAATTG (MfeI restriction site) sequence has been introduced.
(0.70 MB TIF)

Figure S4 Influence of induced transcription on GAA·TTC repeat stability. (A) PCR amplification of a (GAA·TTC)_176_ insert at W0 and at W3 under basal (−Dox) and induced (+Dox) transcription. Induced transcription results in a modest but reproducible decrease in expansion rate among GAA·TTC repeat inserts. (B) 10 week time-course analysis of a (GAA·TTC)_352_ insert under basal and induced transcription. Prolonged culturing during induced transcription leads to an increase in repeat contraction over time.
(1.91 MB TIF)

Figure S5 (A) Modified tandem reporter construct containing the poly(A) site with 400 bp of surrounding sequence (HBB1) from the *HBB* gene inserted upstream of a (GAA·TTC)_176_ insert sequence. (B) Analysis of transcription rates through the GAA·TTC repeat insert sequences in the TAN and HBB1 constructs. Successful transcription through the repeat inserts is expressed as the ratio of hRLUC/FLUC luciferase reporters located in the tandem constructs. Values are normalized to the TAN control construct. The error bars indicate the SEM for an *n* = 3. (C) PCR analysis of GAA·TTC expansion in the TAN and HBB1 constructs. (GAA·TTC)_176_ inserts were sized at W0 and W3. TAN primers add 438 bp to the GAA·TTC insert. HBB1 primers add 448 bp to the GAA·TTC insert. M: 1 Kb plus size standard.
(2.06 MB TIF)
