# Supplementary figures and images for: Progressive GAA·TTC Repeat Expansion in Human Cell Lines
Source: PLoS Genet. 2009 Oct 30;5(10):e1000704. doi: 10.1371/journal.pgen.1000704 (PMC2760145; doi:10.1371/journal.pgen.1000704)

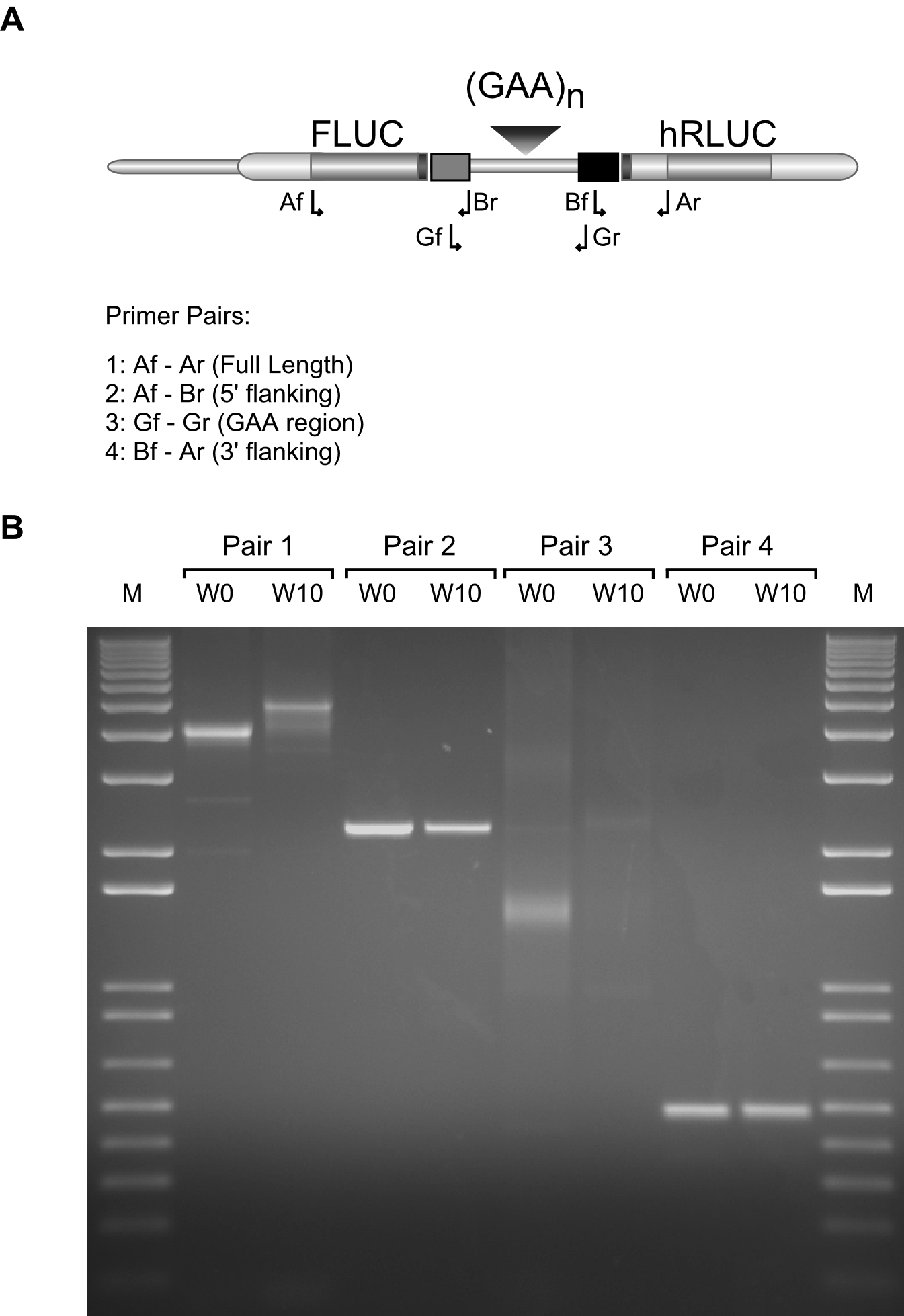

Supplement: Figure S1 — PCR analysis of GAA·TTC insert region and flanking sequence stability. PCR amplification was performed on the genomic samples used in Figure 1 at W0 and W10. Primer pairs were designed to amplify separate regions of the integrated tandem reporter construct. Pair 1 amplifies from the 5′ FLUC region to the 5′ hRLUC region (5′: 2347 bp+(GAA)n+668 bp: 3′). Pair 2 amplifies the 5′ region flanking the GAA·TTC insert (2272 bp sequence beginning 76 bp upstream of GAA·TTC insert). Pair 3 amplifies the GAA·TTC insert sequence (5′: 151 bp+(GAA)n+76 bp: 3′). Pair 4 amplifies the 3′ region flanking the GAA·TTC insert (485 bp sequence beginning 179 bp downstream of GAA·TTC insert). (2.63 MB TIF) [file pgen.1000704.s001.tif]

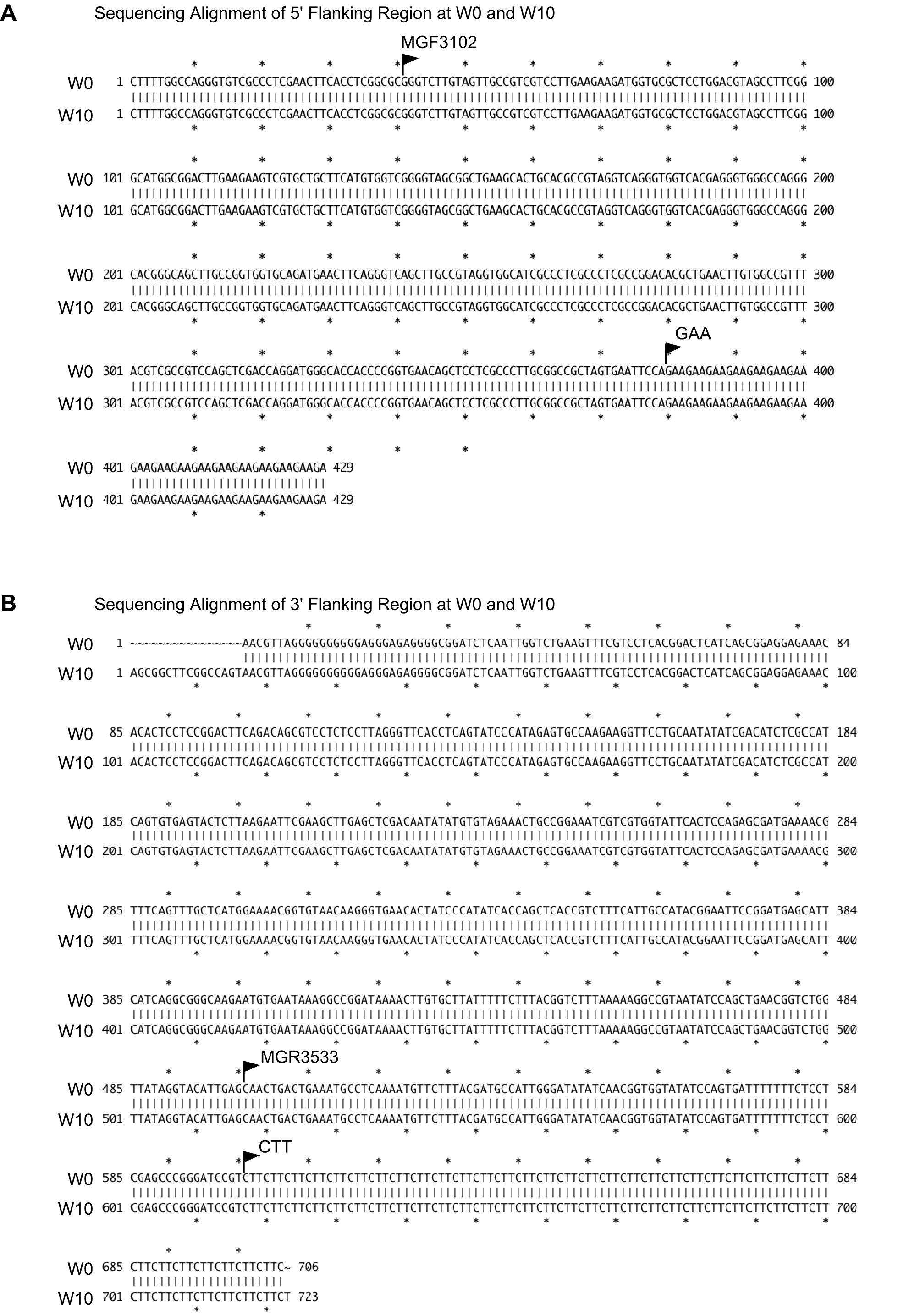

Supplement: Figure S2 — Sequencing analysis of the immediate flanking region surrounding the GAA·TTC repeat insert. Sequencing analysis was performed on the genomic samples used in Figure 1 at W0 and W10. (A) Sequencing alignment of the 5′ flanking region at W0 and W10. (B) Sequencing alignment of the 3′ flanking region at W0 and W10. The locations of the primers used for PCR analysis in Figure 1 (MGF3102 and MGR3533) and the junctions between the flanking region and the repeat insert are shown. (5.73 MB TIF) [file pgen.1000704.s002.tif]

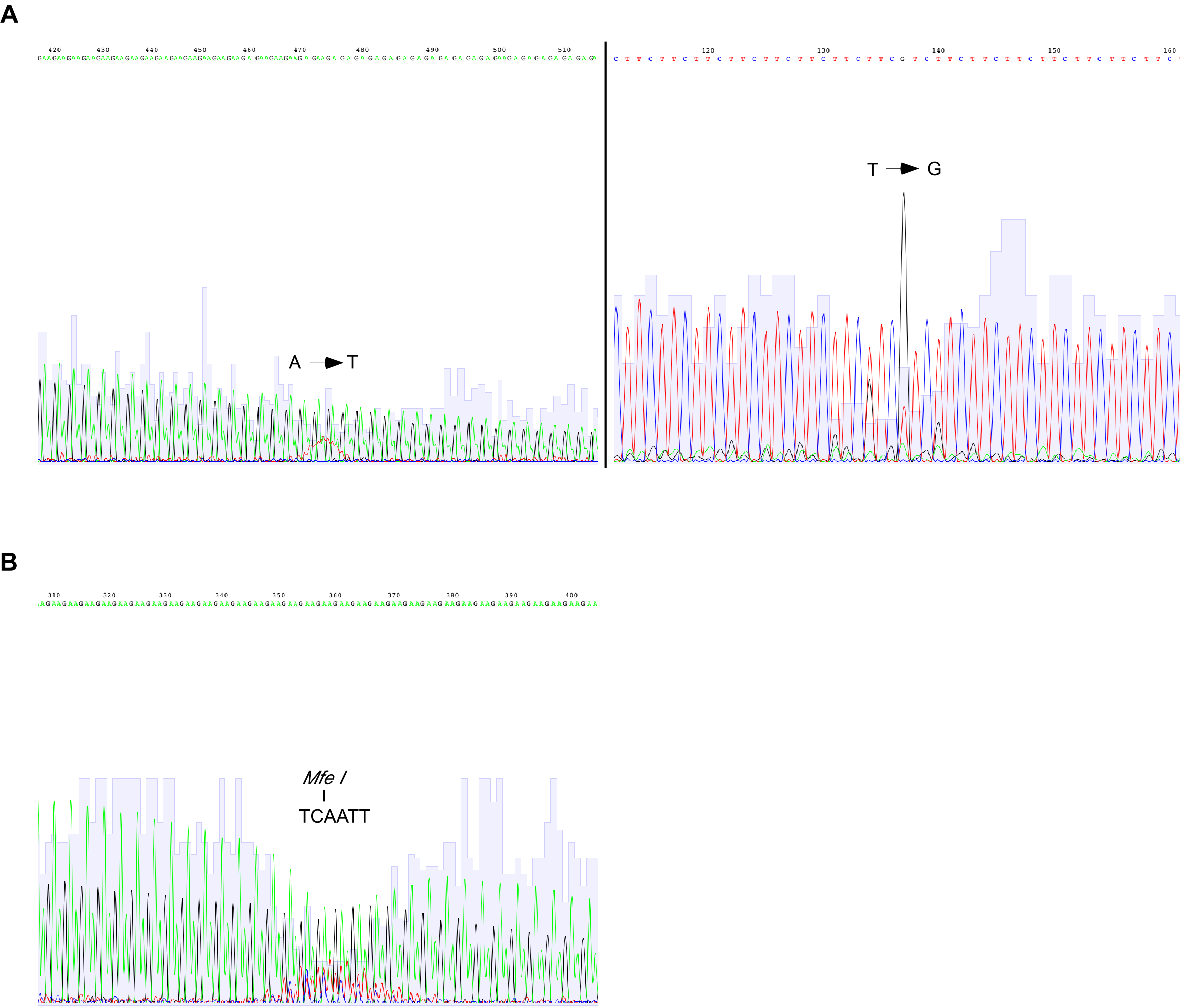

Supplement: Figure S3 — Sequencing data of GAA·TTC repeat region with interrupting mutations. (A) GAA·TTC repeat region from a clone with 2 point mutations within the repeat region. The left panel is from the 5′ - GAA orientation showing an A→T mutation 118 triplets into the repeat region. The right panel is from the 5′ CTT orientation showing an T→G mutation 31 triplets into the repeat region. (B) GAA·TTC region from a clone in which a TCAATTG (MfeI restriction site) sequence has been introduced. (0.70 MB TIF) [file pgen.1000704.s003.tif]

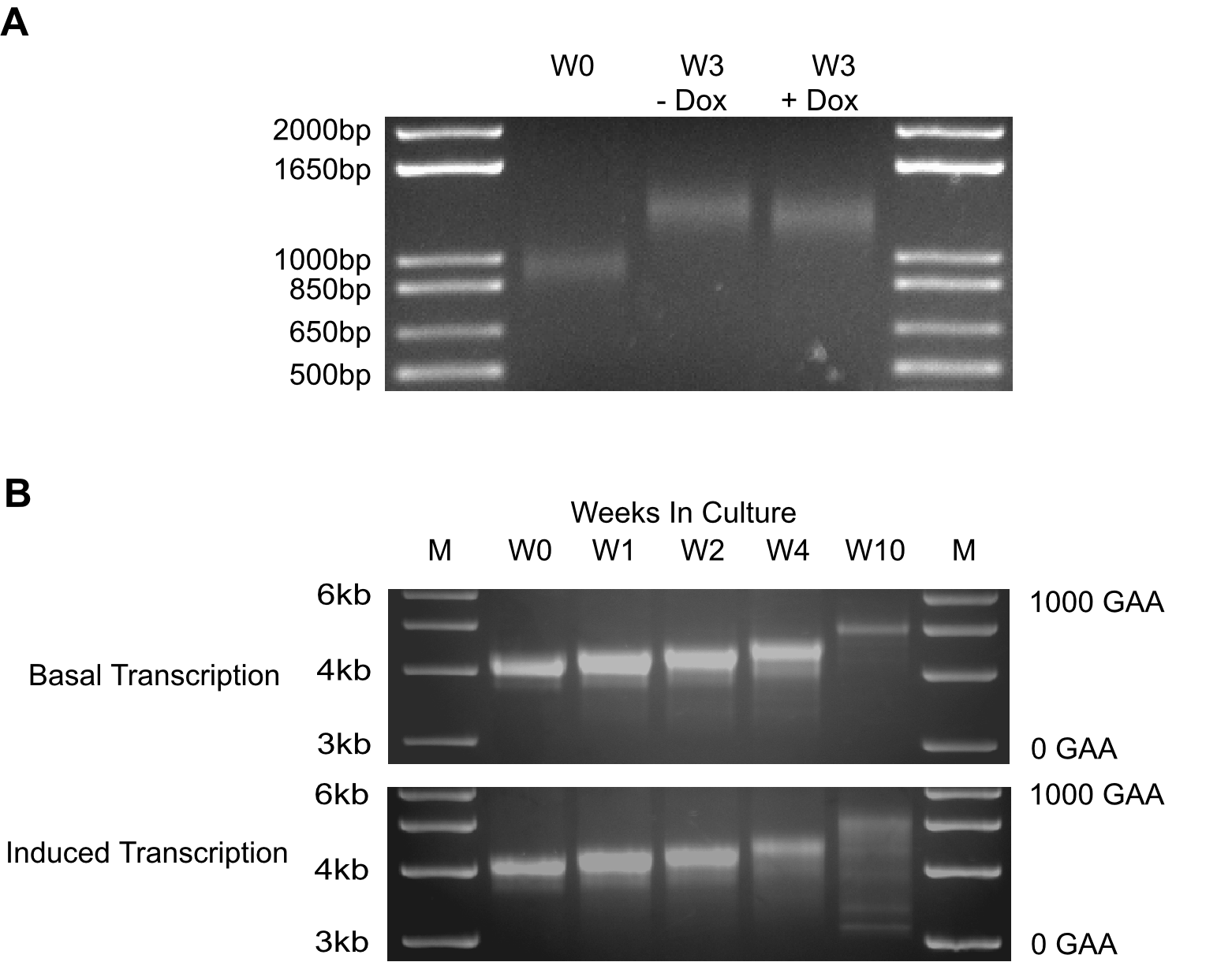

Supplement: Figure S4 — Influence of induced transcription on GAA·TTC repeat stability. (A) PCR amplification of a (GAA·TTC)176 insert at W0 and at W3 under basal (−Dox) and induced (+Dox) transcription. Induced transcription results in a modest but reproducible decrease in expansion rate among GAA·TTC repeat inserts. (B) 10 week time-course analysis of a (GAA·TTC)352 insert under basal and induced transcription. Prolonged culturing during induced transcription leads to an increase in repeat contraction over time. (1.91 MB TIF) [file pgen.1000704.s004.tif]

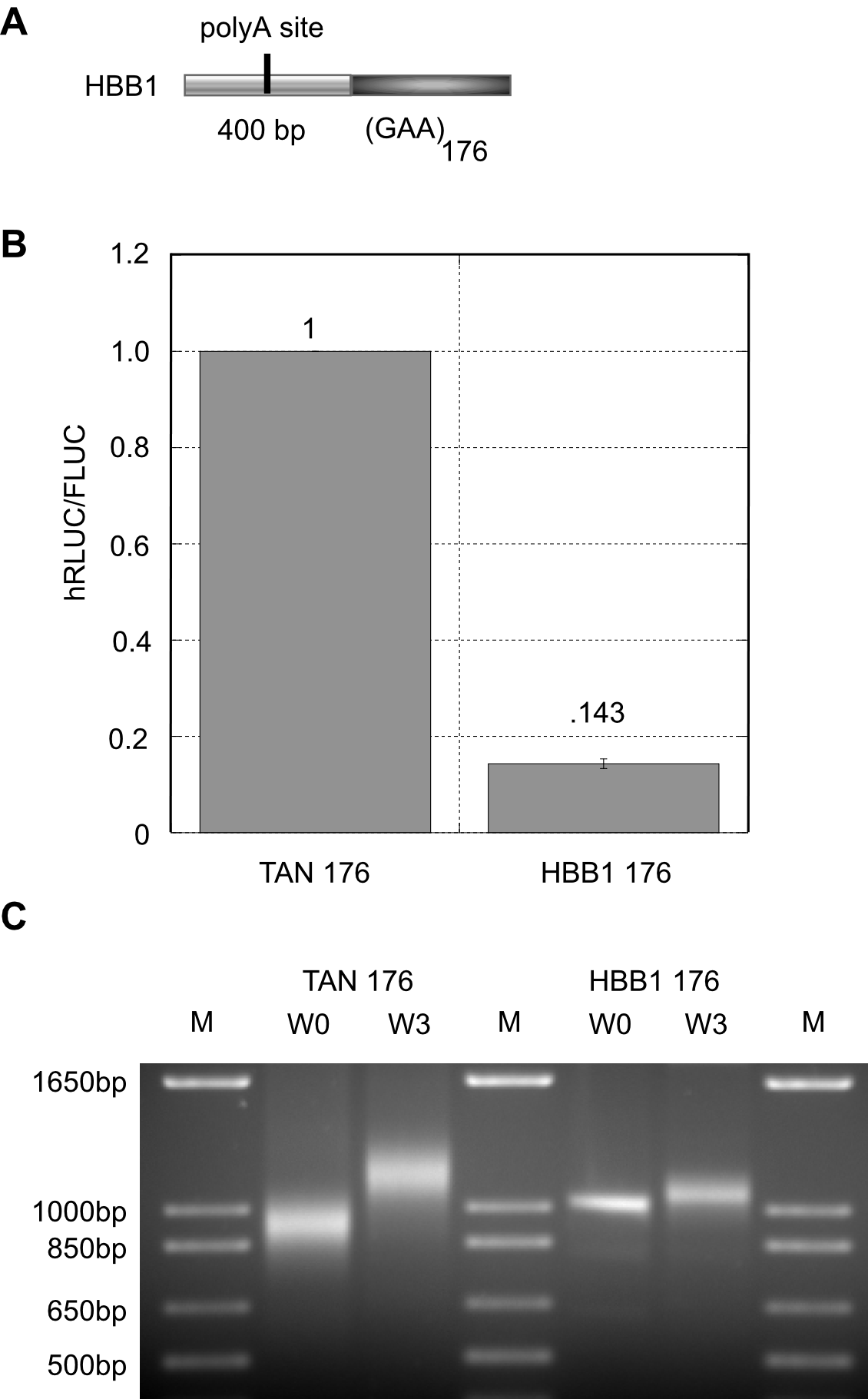

Supplement: Figure S5 — (A) Modified tandem reporter construct containing the poly(A) site with 400 bp of surrounding sequence (HBB1) from the HBB gene inserted upstream of a (GAA·TTC)176 insert sequence. (B) Analysis of transcription rates through the GAA·TTC repeat insert sequences in the TAN and HBB1 constructs. Successful transcription through the repeat inserts is expressed as the ratio of hRLUC/FLUC luciferase reporters located in the tandem constructs. Values are normalized to the TAN control construct. The error bars indicate the SEM for an n = 3. (C) PCR analysis of GAA·TTC expansion in the TAN and HBB1 constructs. (GAA·TTC)176 inserts were sized at W0 and W3. TAN primers add 438 bp to the GAA·TTC insert. HBB1 primers add 448 bp to the GAA·TTC insert. M: 1 Kb plus size standard. (2.06 MB TIF) [file pgen.1000704.s005.tif]
